# Epidemiological association of *Campylobacter jejuni* groups with pathogenicity-associated genetic markers

**DOI:** 10.1186/1471-2180-12-171

**Published:** 2012-08-08

**Authors:** Andreas E Zautner, Carolin Ohk, Abdul Malik Tareen, Raimond Lugert, Uwe Groß

**Affiliations:** 1 Universitätsmedizin Göttingen, Abteilung für Medizinische Mikrobiologie, Kreuzbergring 57, D-37075 Göttingen, Germany

**Keywords:** *Campylobacter jejuni*, Subgroups, fucP, cj0178, *cj0755*/*cfrA*, LOS class, Flagellin glycosylation, Virulence

## Abstract

**Background:**

*Campylobacter jejuni*, the most leading cause for bacterial gastroenteritis worldwide, shows a high genetic diversity among its isolates. Recently, we demonstrated the existence of six *C. jejuni*-groups by combining MLST with six genetic markers. These groups were further characterized by the detection of *cj1321-cj1326*, *fucP*, *cj0178*, *cj0755*/*cfrA, ceuE*, *pldA*, *cstII*, and *cstIII* in order (I.) to show further associations between these different genetic markers and MLST CCs. Moreover, different studies were able to associate several of these markers: a sialylated lipoologosaccharide (*cstII/III*^+^), the gamma-glytamyl-transpeptidase (*ggt*^+^), and the absence of a certain allele of the enterochelin-uptake-binding-protein (*ceuE*_11168_^-^) with severe campylobacteriosis, bloody diarrhea and unpleasant outcome. Additionally more than half of human *Campylobacter-*isolates were assigned to a non-livestock clade associated with the absence of *cj1321-cj1326*. These isolates were considered as mere colonizers.

From the combination of marker genes, the ratio of human isolates in a specific group, and clinical data (II.) it should be demonstrated to which of the previous defined groups these *Campylobacter*-subpopulations, associated with higher virulence, correspond.

**Results:**

Besides the marker gene *pldA*, all new estimated genetic markers show significant differences in their distribution among the various MLST-based groups. Especially the genes for *cj1321-cj1326*, *fucP*, *cj0178*, *cj0755*/*cfrA* are widely associated with each other and split the study population into two major and seven intermediate groups substantiating the previous group-definition, whereas *cstII* and *cstIII* indicate at least three groups following an independent distribution pattern.

**Conclusions:**

Based on these data a group of *C. jejuni*-isolates characterized by the presence of *ansB, dmsA*, *ggt,* and the absence of *cj1365c*, *cj1585c*, *cj1321-cj1326, fucP*, *cj0178*, *cj0755*/*cfrA,* and *cstII/III* was associated with a higher prevalence in human campylobacteriosis, bloody diarrhea as well as hospitalization and bears obviously a higher virulence for humans. In contrast to that better livestock-adapted groups characterized by the ability to utilize L-fucose and the presence of all of the five identified putative *C. jejuni* iron-uptake systems as well as *cj1321-cj1326*, *cj1365c, cj1585c*, and *cstII* and/or *cstIII* (sialylated lipoologosaccharide) is more prevalent in animal hosts and was secondary associated with less severe campylobacteriosis.

## Background

The Gram-negative *Epsilonproteobacterium Campylobacter jejuni*, which is due to recent epidemiological data the most leading cause for bacterial gastroenteritis and Guillain-Barré-syndrome (GBS) worldwide, shows a high genetic diversity among its isolates [1]. As consequence of this genetic and phenotypic diversity several *C. jejuni* subpopulations could be identified on the basis of the presence of non-ubiquitous genes [2]. In a previous study we could identify six *C. jejuni* groups combining multilocus sequence typing (MLST) with six genetic markers: *ansB, dmsA,ggt*, *cj1585c*, *cj1365c* and dimeric *tlp7* (Tlp7_m_ + Tlp7_c_) [2]. Here we could in particular demonstrate that the genes *ansB, dmsA,ggt* occur together in a specific *cj1585c*- and *cj1365c*–negative isolate group [2].

Several studies were able to correlate further genetic markers with clinical parameters. Thus, the question was addressed how a sialylated lipoologosaccharide (LOS) affects the severity of the *Campylobacter*-trigged diarrhea [3-5]. It was demonstrated that a sialylated LOS of the *Campylobacter* cell wall is associated with an increased occurrence of bloody diarrhea and a longer duration of symptoms [3-5].

Champion and coworkers made a further interesting finding. They demonstrated that 55.7% of *C. jejuni* isolates from human faeces belong to a non-livestock clade that misses the flagellin *O*-glycosylation cluster encoded by the genes *cj1321-cj1326*[6]. *Cj1321-cj1326*-negative strains originate mostly from asymptomatic carriers and the environment. Thus, flagellin *O*-glycosylation may play as well a role in cell invasion, and in consequence for the virulence in humans.

Another study of Feodoroff and coworkers identified a *C. jejuni*-subpopulation in which they were able to detect the gamma-glytamyl-transpeptidase gene (*ggt*) but not the fucose permease gene (*fucP*), the phospholipase A gene *(pldA)* and the enterochelin-uptake-binding-protein gene (*ceuE*) using *pldA-* and *ceuE*-primers derived from the NCTC 11168 genome sequence (The corresponding genes are designated in the following as *pldA*_11168_ and *ceuE*_11168_) [7]. These isolates could be associated with a higher rate of hospitalizations and bloody diarrhea [7].

To determine the distribution of these further genetic markers as well as their association with *ansB, dmsA,ggt*, *cj1585c*, *cj1365c* and dimeric *tlp7* and secondary their correlation with the clinical data of the above mentioned studies, we further characterized the same 266 isolates by screening for the presence of eight additional genetic markers: the flagellin *O*-glycosylation locus *cj1321-cj1326*[6], the L-fucose permease gene *fucP*[8], the outer membrane siderophore receptor *cj0178*[9,10], the iron uptake protein/ferric receptor *cj0755*/*cfrA*[9,10], the enterochelin uptake binding protein *ceuE*[11], the outer membrane phospholipase A *pldA*[12], as well as the lipooligosaccharide sialyltransferases *cstII* and *cstIII*[13,14]*.*

## Results

**Figure 1:**
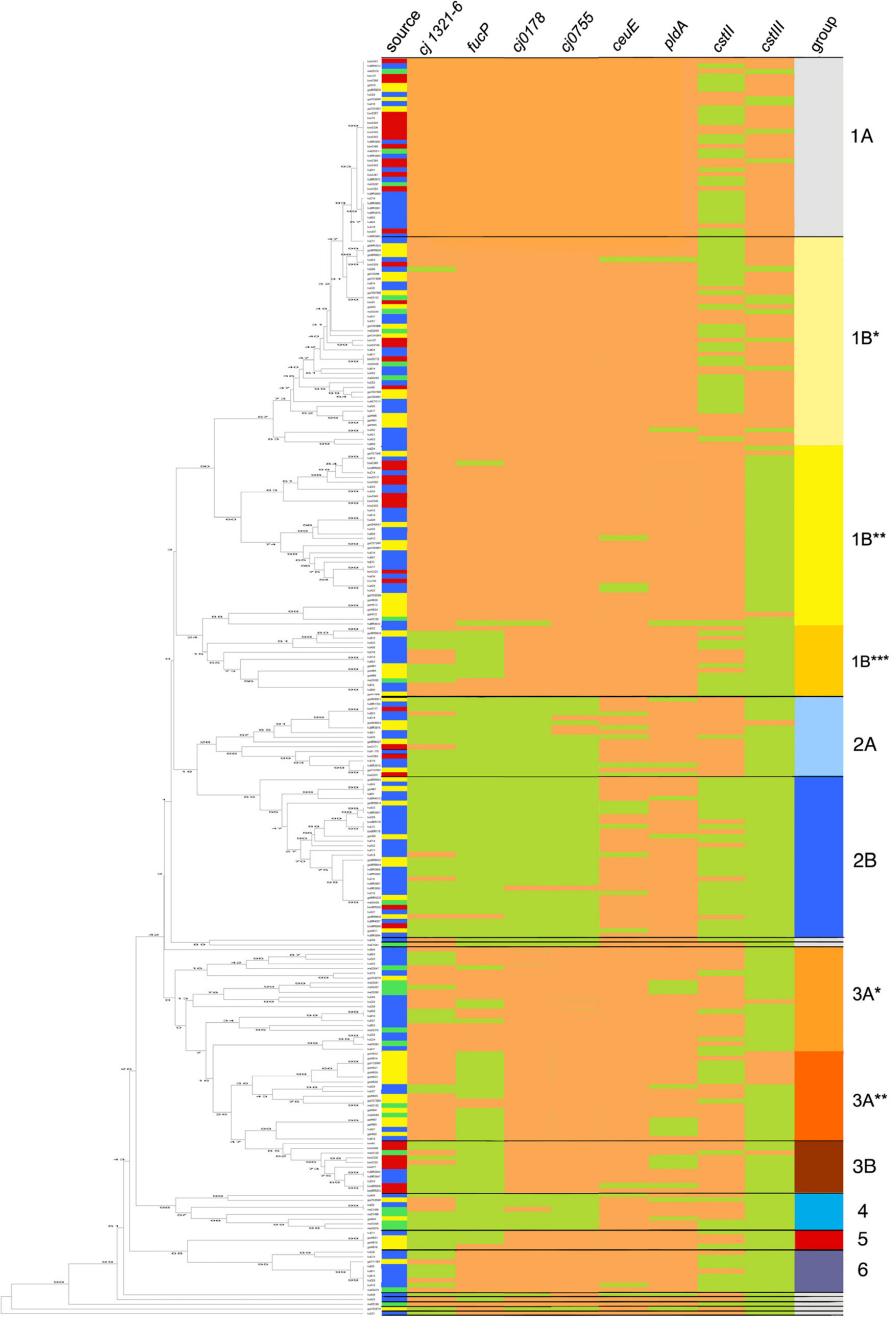
**MLST-sequence based UPGMA-tree and the arrangement of the six different marker genes within the six defined groups (twelve subgroups).** On the left side the MLST-sequence based UPGMA-tree of 266 *C. jejuni* isolates is depicted. The numbers shown on the branches of the tree indicate the linkage distances. The right side of the table lists all isolates in the order of the UPGMA-tree depicting the source of the isolate, the presence or absence of the six marker genes and their belonging to one of the groups listed in Table1. Source: Human isolates are marked blue, chicken isolates yellow, bovine isolates red, and turkey isolates green. Marker genes: Presence of a genetic marker is marked with a light red shade, absence with a light green shade. The marker genes from left to right are: *cjj1321-6***:***O*-linked flagellin glycosylation locus; *fucP*: L-fucose permease gene (*cj0486*); *cj0178*: outer membrane siderophore receptor; *cj0755*: iron uptake protein (ferric receptor *cfrA*); *ceuE*: enterochelin uptake binding protein; *pldA*: outer membrane phospholipase A; *cstII*: LOS sialyltransferase II; *cstIII*: LOS sialyltransferase III; The last column gives the group according to Table1: light grey (1A), light yellow (1B^*^) intense yellow (1B^**^), dark yellow (1B^***^) cyan blue (2A), bondi blue (2B), carrot-orange (3A^*^), orange-red (3A^**^); rust-red (3B), turquoise [4], red [5], steel-blue [6] and white (singeltons).

The frequency of all eight new determined genetic markers in all tested 266 isolates and in each subgroup is listed in Table1. Additionally the ratio of human isolates as parameter for the clinical relevance of the particular isolate group is listed there. A pictorial representation of the marker gene distribution among the various subgroups as well as their isolate origin is shown in Figure1.

**Table 1:** Distribution and association of genetic markers, LLC and MLST-CC within the determined subgroups

**(sub-) group**	**No. of isolates with marker gene/total no. (%)**									***human origin***
	***cj1321-1326***	***fucP***	***cj0178***	***cj0755***	***ceuE***_**11168**_^**1**^	***pldA***_**11168**_^**2**^	***cstII***	***cstIII***	**LLC**^**3**^	
**1a**	**38/38**^**#**^**(100)**	**38/38**^**#**^**(100)**	**38/38**^**#**^**(100)**	**38/38**^**#**^**(100)**	**38/38**^**#**^**(100)**	**38/38**^**#**^**(100)**	13/38^°^(34.2)	**33/38**^**#**^**(86.4)**	C/A	16/38(42.1)
1b^*^	**43/44**^**#**^**(97.7)**	**44/44**^**#**^**(100)**	**44/44**^**#**^**(100)**	**44/44**^**#**^**(100)**	**42/44**^**°**^**(95.5)**	**41/44(93.2)**	16/44^°^(36.4)	**37/44**^**#**^**(84.1)**	C/A/B	19/44(43.2)
1b^**^	**38/38**^**#**^**(100)**	**36/38**^**#**^**(94.7)**	**37/38**^**#**^**(97.4)**	**38/38**^**#**^**(100)**	**35/38(92.1)**	**37/38**^**°**^**(97.4)**	**37/38**^**#**^**(97.4)**	2/38^#^(5.3)	B2	19/38(50.0)
1b^***^	7/15(46.7)	5/15^°^(33.3)	**15/15**^**#**^**(100)**	**15/15**^**#**^**(100)**	**14/15**^**#**^**(93.3)**	**15/15**^**#**^**(100)**	6/1z(40.0)	0/15^#^(0.0)	B, D	**9/15(60.0)**
**2a**	2/17^#^(11.8)	0/17^#^(0.0)	0/17^#^(0.0)	3/17^#^(0.0)	12/17(70.6)	14/17(82.4)	**16/17**^**#**^**(94.1)**	1/17^#^(5.9)	A1/B	8/17(47.1)
**2b**	3/34^#^(8.8)	1/34^#^(2.9)	1/34^#^(2.9)	1/34^#^(2.9)	26/34^°^(76.5)	29/34(85.3)	5/34^#^(14.7)	0/34^#^(0.0)	D/E/H/U	**22/34**^°^**(64.7)**
3a^*^	**15/22(68.2)**	**18/22**^**°**^**(81.8)**	**22/22**^**#**^**(100)**	**22/22**^**#**^**(100)**	18/22(81.8)	18/22(81.8)	**18/22**^**#**^**(81.8)**	1/22^#^(4.5)	-	**15/22**^°^**(68.2)**
3a^**^	**16/19**^**°**^**(84.2)**	2/19^#^(10.5)	**19/19**^**#**^**(100)**	**19/19**^**#**^**(100)**	**18/19**^**#**^**(94.7)**	11/19(57.9)	**12/19(63.2)**	**7/19(36.8)**	E	4/19^°^(21.1)
**3b**	2/11^°^(18.2)	0/11^#^(0.0)	**11/11**^**#**^**(100)**	**11/11**^**#**^**(100)**	**10/11(90.9)**	8/11(72.7)	**10/11(90.9)**	1/11(9.1)	-	3/11(27.3)
**4**	3/8(37.5)	0/8^#^(0.0)	1/8^#^(12.5)	0/8^#^(0.0)	**7/8(87.5)**	6/8(75.0)	**5/8(62.5)**	0/8^#^(0.0)	-	2/8(25.0)
**5**	0/4^#^(0.0)	1/4(25.0)	**4/4**^**#**^**(100)**	**4/4**^**#**^**(100)**	**4/4**^**#**^**(100)**	**4/4**^**#**^**(100)**	2/4(50.0)	0/4^#^(0.0)	-	1/4(25.0)
**6**	3/9(33.3)	**9/9**^**#**^**(100)**	**9/9**^**#**^**(100)**	**9/9**^**#**^**(100)**	**8/9(88.8)**	**8/9(88.8)**	2/9^°^(22.2)	0/9^#^(0.0)	A/D	**7/9(77.8)**
**all**	170/266(63.9)	154/266(57.9)	204/266(76.7)	208/266(78.2)	232/266(87.2)	229/266(86.1)	142/266(53.4)	82/266(30.8)	all	128/266(48.1)

**Marker genes***cjj1321-cj1326***:***O*-linked flagellin glycosylation locus; *fucP*: L-fucose permease gene (*cj0486*); *cj0178*: outer membrane siderophore receptor; *cj0755*: iron uptake protein (ferric receptor *cfrA*); *ceuE*: enterochelin uptake binding protein; *pldA*: outer membrane phospholipase A; *cstII*: LOS sialyltransferase II; *cstIII*: LOS sialyltransferase III; LLC: lipooligosaccharid (LOS) locus class; MLST-CC: Multilocus sequence typing clonal complex; **footnotes**: ^1^negative corresponds to only detectable using the forward primer based on the 81-176 genome sequence: 5’-GAT-AGA-GTC-GCA-GGC-GTT-CC-3’^+2^negative corresponds to only detectable using the forward primer based on the 81-176 genome sequence: 5’-AAA-CTT-ATG-CGT-TTT-T-3’ ^3^LLC according to Habib *et al.* 2009 [3], Hotter *et al.* 2010 [15], Revez *et al.* 2011 [16]; p<0.05/^#^ p<0.001 significance level in comparison to the remaining isolates belonging not to the corresponding group, additionally the values in subgroups with above average numbers of positive isolates are given in bold numbers; in the case of *ceuE* and *pldA* the NCTC 11168 typical allele presence is given in bold if the isolate numbers were above average.

The flagellin *O*-glycosylation locus *cj1321-cj1326* as marker for livestock-associated strains could be detected in the majority of the isolate groups: 1A, 1B*, 1B**, 3A and 4, assuming their livestock association. In contrast to that, especially the groups 2A + B as well as 1B***, 3B and 5 were negative for this marker gene.

A comparable distribution pattern could be demonstrated for the *fucP* gene. The isolate groups 1A, 1B*, 1B**, 3A* and 6, are positive for this marker gene, whereas the *fucP* genes was nearly absent in the groups 1B***, 2A + B, 3A** + B and 4.

Feodoroff and coworkers identified a subpopulation in which they were not able to detect *ceuE* using *ceuE*-primers derived from the NCTC 11168 genome sequence [7]. The same phenomenon was described by them for *pldA* using NCTC 11168 genome based primers, but here the differences were not significant [7]. Using additional forward primers based on the gnome sequence of the 81–176 strain (see Table2), we could detect *ceuE* and *pldA* in the whole test population. Using exclusively the NCTC 11168 genome based primers a significant lowered *ceuE*-detection rate was only observerd for group 2 isolates (24.0%, p < 0.002). There were no significant differences in the *pldA* detection using additional 81–176 genome-based primes in our study population. 

**Table 2:** Primer

**Gene**	**Primer name**	**Sequence 5’-3’**	**Annealing temp**	**Reference**
***cj0178***	*cj0178*-F01	TGTAGGCGGGGGTGGCAAGA	54.0°C	this study
	*cj0178*-R01	ACGACCGCGAGCAGAATTGC		
***cj0755/cfrA***	*cj0755*/cfrA-F01	ATGGCCGCGAAGTCGTAGGG	54.0°C	this study
	*cj0755*/cfrA-R01	AGCGATCTATTTGCCACTCGCCT		
***cj1321***	CjNCTC11168-1321_f	AAAATGTCATCATCATAGGAGCG	60.0°C	[6]
	CjNCTC11168-1321_r	TCTAAGTTTACGCAAGGCAACA		
***cj1322***	CjNCTC11168-1322_f	GACTTTGGTTTAATGGGTAAGCA	59.6°C	[6]
	CjNCTC11168-1322_r	TTCCGGCGTTAAAATTAGAAAA		
***cj1323***	CjNCTC11168-1323_f	AGAACGATTTACCCCATTGAAA	59.7°C	[6]
	CjNCTC11168-1323_r	ATTTGCTAAAGCTCCTCGATTG		
***cj1324***	CjNCTC11168-1324_f	TGCCGTAAGTGGAGGTAAAGAT	60.0°C	[6]
	CjNCTC11168-1324_r	TCTGCACACATTGTTCTATCCC		
***cj1325***	CjNCTC11168-1325_f	ACGGATTACTTTTTCCAGATGGT	60.0°C	[6]
	CjNCTC11168-1325_r	TTTGCTTTGAAAATACGCTGAA		
***cj1326***	CjNCTC11168-1326_f	TACATTTCATCGATAAAGCCGA	59.7°C	[6]
	CjNCTC11168-1326_r	AAATATAATGGTGTGCCGATCC		
***fucP***	cj0486FWD	GATAGAGCATTAAATTGGGATG	52.0°C	[8]
	cj0486REV	CCTATAAAGCCATACCAAGCC		
	rpoC	GAACTTGCTATTGCTGAGCC		
	rpsL	ACCCTAGTGCAAACTCCCCT		
***ceuE***	ceuE-81176F01	GATAGAGTCGCAGGCGTTCC	60°C	this study
	ceuE405F	GATAAAGTCGTTGGCGTTCC		[7]
	ceuE405R	GCGAGATTGGAGGACCAAAGG		
***pldA***	pldA-81178F01	AAACTTATGCGTTTTT	45°C	this study
	pldA-84fwd	AAGCTTATGCGTTTTT		[7]
	pld-981rev	TATAAGGCTTTCTCCA		
***cstII***	orf7ab	ACTACACTTTAAAACATTTAATCC AAAATCA	56°C	[14]
	orf7ab	CCATAAGCCTCACTAGAAGGTATGAGTATA		
***cstIII***	orf7c	TTGAAGATAGATATTTTGTGGGTAAA	56°C	[14]
	orf7c	CTTTAAGTAGTGTTTTATGTCACTTGG		

Furthermore, we included the genes *cj0178*, an outer membrane siderophore receptor, and *cj0755*, an iron uptake protein (ferric receptor), in the panel of marker genes. The gene products of *cj0178* and *cj0755* are like enterochelin, CeuE, involved in the microbial iron uptake. Thus, it was, because of their functional association to CeuE, suggestible that they may be associated with bloody diarrhea like *ceuE*[7] as well. Both genes could be detected, mostly associated with each other, in more than 76% of all isolates. In the groups 2 (A + B) and 4 they are nearly completely absent, whereas about 100% of the remaining groups are positive for both genes.

Additionally, we looked for the presence of *cstII* and *cstIII* in order to distinguish isolates with sialylated LOS from isolates with non-sialylated LOS. There are already more detailed studies associating MLST CC with certain LCC [3,15,16] allowing us to associate a particular isolate group with specific LCC only on the basis of the MLST-CC and the information about the absence or presence of *cstII* and *cstIII* (see Table1 and Figure1). Group 1A and 1B* were generally tested positive for *cstIII*. The subgroup named 1B**, which is comprised of CC 48 and CC 206 isolates, is only *cstII* but not *cstIII* positive. Isolates from the subgroup 1B*** (CC 49 and CC 446) are partially positive, partially negative for *cstII* but generally *cstIII*-negative. All in all, 23 isolates are positive for *cstII* and *cstIII*. Most of these double-positive isolates belong to group 1 (87.0%) and CC 21 (65.2%).

The isolates of group 2A are in the majority *cstII*-positive, in contrast to group 2B isolates that are negative for both, *cstII* and *cstIII*, which means that these isolates bear a non-sialylated LOS. Most of the group 3 isolates are positive for *cstII* but not *cstIII*, besides a minority of CC 353 isolates that are *cstIII*-positive. The majority of isolates in the groups 4, 5, and 6 are *cstII*- and *cstIII*-negative (non-sialylated LOS).

Finally the ratio of human isolates in comparison to all animal isolates was significantly (p = 0.04355) increased in the *ggt*-positive subgroup 2B, whereas the difference for the whole group 2 (A + B) was increased but not significant. An increased ratio of human isolates could be also detected for the *fucP*-negative subpopulation (p(1B*** + 2) = 0.04790) as well as the *ceuE*-negative (referring to a PCR using NCTC 11168-based primers) subpopulation (p(2 + 3A*) = 0.00825). However, we could not detect any significant association between a particular animal host species and the presence of the eight tested genetic markers (results not shown). With the exception of group 1B** with a significant (p = 0.01374) lower hospitalization rate and group 3A* with a significant (p = 0.00020) lower rate of bloody diarrhea no significant differences in the clinical parameters could be detected within this study population.

## Discussion

Looking at all detected genetic markers we could describe two major types of marker gene combinations represented by group 1A and group 2B. All other groups depict a gradual transition of marker gene combinations between these two groups. Thus the main focus on attention should be on these two groups. Group 1A is characterized by the presence of *cj1365c*, *cj1585c*, *dimeric tlp7*[2], *cj1321- cj1326, fucP, cj0178*, *cfrA/cj755, and ceuE*_*11168*_ as well as the absence of *ansB, dmsA*, *ggt* and *cstII*. In contrast to that, group 2B is an inverted mirror image of this constellation: positive for *ansB, dmsA*, *ggt* but negative for *cj1365c*, *cj1585c*, *dimeric tlp7*[2], *cj1321- cj1326, fucP, cj0178*, *cfrA/cj755, ceuE*_*11168*_ as well as *cstII/III*.

Champion and coworkers identified the flagellin *O*-glycosylation locus *cj1321-cj1326* as marker present in livestock-associated strains, whereas 55.7% of clinical isolates were shown by them to be negative for this gene cluster [6]. According to their data, *cj1321-cj1326*-negative strains originate mostly from asymptomatic carriers and the environment [6]. Due to our data, 63.9% of the tested *C. jejuni* isolates show livestock association based on the presence of *cj1321-cj1326.* But in contrast to their findings the non-livestock-associated group 2B is significantly more often associated with human origin and thus, bears obviously higher pathogenic potential for humans than the livestock-marker positive strains.

The *fucP* gene was shown to be present only in isolates negative for *ggt*[8], which is in accordance with our findings. The *ggt*-positive group 2 is almost completely free of *fucP*-positive isolates. Interestingly, group 6 isolates, positive for the *ggt*-associated marker genes *ansB* and *dmsA* but not for *ggt*, are mostly able to utilize L-fucose. The *fucP* distribution pattern is similar to that of the livestock-association marker genes *cj1321-cj1326* and the serine protease Cj1365 [2]. Thus, *fucP* should be considered as a further marker for livestock association. It can be suggested that the fucose permease is a crucial prerequisite for dwelling in the mucosa layer, while it enables the bacterial cell to metabolize mucosal L-fucose.

The ability to acquire iron is an essential prerequisite for bacterial replication and thus an important virulence factor especially in iron restricted environments [17,18]. While *C. jejuni* has no own siderophores [10] it makes use of exogenous siderophores produced by accompanying bacterial species [19]. At all five different iron uptake systems have been detected in the genome of *C. jejuni* NCTC 11168 [10], but the genome sequence of strain 81–176 reveals three fundamental differences in this regard [9]. Cju15, a protein of unknown function, replaces the gene *cfrA/cj0755*, which encodes a ferric uptake receptor [9]. A second iron uptake transport system encoded by the genes *cj0173c-cj0182* is missing critical components e.g. *cj0178* and *tonB3*[9], and in the gene cluster encoding the enterochelin uptake system *cju30* is inserted between *cj1355* and *cj1356c*[9]. Additionally the enterochelin uptake system (CeuBCDE; Cj1352 to Cj1355) is ubiquitous within the *C. jejuni* population, but it shows sequence variability detectable by PCR using different primers. A *C. jejuni* subpopulation, associated with a higher rate of bloody diarrhea requiring hospitalization, was identified by Feodoroff and coworkers [7]. This subpopulation was positive for *ggt*, but *ceuE* was not detectable using *ceuE*-primers derived from the NCTC 11168 genome sequence. This subpopulation corresponds to group 2 in our scheme. In a significant number of group 2 isolates it was only possible to detect the ubiquitous gene for *ceuE* using primers derived from the genome sequence of *C. jejuni* strain 81–176 (for *pldA* we detected no significant differences). In this group of isolates the iron uptake system components *cj0178* and *cfrA/cj755* are absent in nearly 100% of the isolates. Thus, the two groups identified by Feodoroff *et al.* associated with bloody stools/GGT-production and an increased hospitalization rate/*ceuE*_11168_-presence overlap to a larger part that corresponds to group 2B. Besides *ggt* and *ceuE*_11168_, *cj0178* and *cfrA/cj755* should be considered as marker genes correlating with clinical data.

Parker *et al.* defined, according to the organization of the LOS locus, various LOS locus classes (LLC). The LOS locus of LLC A, B, C, M and R includes the sialic acid synthase (*neuBCA*) and two class-specific sialyltransferases: *cstII* in LLC A, B, M, R and *cstIII* in LLC C [19,20]. It was demonstrated that the LOS plays a role for epithelial cell invasion [4] and is associated with the clinical course of gastro-enteritis [5]. In this study, we detected just the key-enzymes for LOS sialylization *cstII* and *cstIII*. Besides the isolates of the groups 2B and 6, the test population was either *cstII* or *cstIII* positive. Group 1A and 1B* isolates were predominantly positive for *cstIII*. This corresponds to the results of Habib *et al.* that CC 21 belongs to either LCC C or LCC A [3]. The subgroup 1B**, consisting of CC 48 and 206 isolates, is only *cstII* but not *cstIII* positive, corresponding mostly to LLC B [3,15]. The isolates of the subgroup 1B*** (CC 49 and CC 446) were demonstrated to be partially positive, partially negative for *cstII* but generally *cstIII*-negative. This corresponds to LLC B and D due to few isolates described by Habib *et al.*[3]. The majority of group 2A isolates was tested positive for *cstII*, corresponding to LCC A1 and B [3,16] in contrast to group 2B isolates that were tested negative for both *cstII* and *cstIII* and belong to LLC D and E(H) [3]. Positive tested for *cstII* but not *cstIII* was the majority of isolates in group 3. An exclusion were the isolates of CC 353 that are *cstIII*-positive (corresponding to LCC C). The negative test result for *cstII*- and *cstIII* of the majority of isolates in the groups 4, 5, and 6 implies that they belong to LLCs with non-sialylated LOS. Hotter *et al.* associated LCC D and E, corresponding to group 2B in our study, with an increased hospitalization rate [5], that is in accordance with the results obtained by Feodoroff and coworkers for the *ggt*-positive and ceuE_11168_-negative group [6] as well as with our prevalence rates for isolates of human origin. In contrast to our data and the data of Feodoroff *et al.*[7] Hotter and coworkers associated LCC B and C with a higher frequency of bloody stools [5]. This group of isolates corresponds for the most part to the group 1 but also 2A.

## Conclusions

In general, the arrangement of the eight additional marker genes and the ratio of isolates of human origin substantiates and complements our prior definition of the subgroups.

One outstanding population formed by the groups 1A + B, which is able to utilize L-fucose, seems to be livestock-adapted due to the presence of *cj1321-cj1326*, *cj1365c* and *cstII* and/or *cstIII*, and has all of the five identified putative iron uptake systems of *C. jejuni*. These strains do not exhibit the genes for an extended amino acid metabolism. Due to their livestock adaptation these isolates are less prevalent in humans and secondarily associated with less severe campylobacteriosis.

Contrary to that, group 2 isolates possess an extended amino acid metabolism (positive for *ansB, dmsA,* as well *ggt*) and are not able to metabolize L-fucose (*fucP*-negative). Group 2 isolates possess only three of five iron uptake systems. This group splits into the two subgroups 2A and 2B. The subgroup 2B is additionally negative for the livestock markers *cj1365c*, *cj1321- cj1326*, as well as *cstII*/*III*. In contrast to that, subgroup 2A is positive for *cj1365c* and *cstII*, but *cj1321- cj1326* is likewise not present. Additionally, subgroup 2A is characterized by the presence of the flagellum-secreted nonflagellar protein A1 encoded by *fspA1*[20]. The remaining subgroups demonstrate a somewhat intermediate marker gene profile compared to 1A and 2B. In this respect, group 6 seems noteworthy, as the corresponding isolates are positive for *ansB* and *dmsA,* typical for group 2 as well as *fucP*, *cj0178*, *cj0755* and *cj1365c* typical for group 1 but not *ggt* or *cj1321- cj1326*. Furthermore, only half of group 6 isolates posses a sialylated LOS.

The high virulent isolate subpopulations identified by Mortensen, who associated LCC D and E with a higher hospitalization rate [5] and these of Feodoroff, who associated *ggt* and a *ceuE* gene, that is not detectable with primers based on the NCTC 11168 sequence, with severe campylobacteriosis and bloody diarrhea [7], seem to overlap at least partially in group 2, with the highest pathogenic potential i.e. the highest virulence for humans. Surprisingly, the asymptomatic colonizers identified by Champion *et al.*[6] and isolates bearing a non-sialylated LOS seem to predominate this high virulent isolate group.

Finally, it should be questioned especially for *cstII/III*, if there is a causal relationship between a particular genetic marker and clinical parameters, while particular genetic markers are associated with each other and the causal relationship to clinical parameters could be due to a causal relation of an associated genetic marker.

## Methods

### *C. jejuni* isolates

A total of 266 *C. jejuni* isolates, 128 of human, 66 of chicken, 45 of bovine, and 27 of turkey origin, with already determined MLS-type and characterized for six genetic markers were selected from our collection [2]. That means about half of the isolates were of human (128) and half of animal (138) origin, what should help to make statements about the clinical relevance of a particular isolate group due to the proportion of isolates originating from human stool samples. The avian and bovine isolates were obtained from the German *Campylobacter* reference center at the *Bundesinstitut für Risikobewertung* (Federal Institute for Risk Assessment) in Berlin, Germany. The human isolates originate from stool samples of hospitalized patients of the University Medical Center Göttingen, Germany (40%) as well as outpatients of several doctor’s offices in the city of Göttingen (60%). For these strains the parameters watery diarrhea (85%) vs. bloody diarrhea (15%) are known. Additionally 42 well-characterized isolates of the CampyNet research network strain collection were included as references. All isolates of this study were PCR-positive for *ciaB* and the *cdtB*.

The *C. jejuni* isolates were cultured on Columbia agar base (Merck) supplemented with 5% sheep blood (BA) and incubated at 42**°**C under microaerophilic conditions (5% O_2,_ 10% CO_2,_ 85% N_2_) for 24 hours prior to DNA extraction.

### DNA extraction and marker gene detection

Genomic DNA of *C. jejuni* was isolated using the QIAamp DNA Mini Kit (Qiagen) according to the manufacturer’s instructions. For detection of the different genetic markers the primers listed in Table2 were used.

### Phylogenetic analysis

For construction of a UPGMA-dendrogram (unweighted-pair group method using average linkages) the MEGA4 software was used [21], and the *C. jejuni* MLST website (http://pubmlst.org/campylobacter/) developed by Keith Jolley and Man-Suen Chan, sited at the University of Oxford was consulted for assignation of sequence types and clonal complexes [22].

### Statistical analyses

Statistical analysis was performed using the Statistica software. The χ²-test was used to test for significant differences/similarities in the frequencies of the various genetic markers within the defined groups. The obtained p-values are indicated in Table1.

## Abbreviations

GBS: Guillain-Barré-syndrome; LOS: lipoologosaccharide; LLC: lipoologosaccharide locus classes; *ggt*: gamma-glytamyl transpeptidase gene; *ceuE*: enterochelin uptake binding protein; *ciaB*: *Campylobacter* invasion antigen B gene; *cdtB*: cytolethal distending toxin subunit B gene; *cj*: gene numbering based on the genome sequence of *Campylobacter jejuni* strain NCTC 11168; *cfrA*: ferric receptor gene in *C jejuni* (iron uptake protein); *fucP*: L-fucose permease gene; *pldA*: outer membrane phospholipase A gene; *cstII/III*: lipooligosaccharide sialyltransferases II and III genes; *ansB*: asparaginase gene with an *N*-terminal *sec-*dependent secretion signal; *dmsA*: dimethyl sulfoxide oxidoreductase subunit A gene; *tlp7*: transducer like protein 7 gene; UPGMA: unweighted-pair group method using average linkages; MLST: multi-locus sequence typing; CC: clonal complex.

## Competing interests

All authors declare no competing interests.

## Authors’ contributions

AEZ conceived the study idea, performed all mathematical analysis and drafted the manuscript, CO performed bacterial culture, DNA isolation and PCR-analysis, AMT performed DNA isolation and MLST-PCR, RL performed DNA sequencing and assisted in drafting the manuscript. UG participated in the study design and helped drafting the manuscript. All authors read, commented and approved the manuscript.
